# Ischemic Stroke as the First Manifestation of Primary Antiphospholipid Syndrome in a Patient With a Recent Diagnosis of HIV: A Case Report

**DOI:** 10.7759/cureus.101853

**Published:** 2026-01-19

**Authors:** Felipe Esparza Salazar, Alma Sofia Mojica López, Maria Fernanda Bautista Gonzalez, Ericka C Loza López, Gabriela Cano Herrera, Enrique de Jesús Guzmán Argüelles, Ana Julieta Ochoa Campos, Gloria Itzel Cerda Hernández, Sandra Caro Timoteo, Rafael Trejo Vázquez, Luis Francisco García Ortega, Monserrath Sánchez Meza

**Affiliations:** 1 Internal Medicine, Centro de Investigación en Ciencias de La Salud (CICSA) Facultad de Ciencias de la Salud, Universidad Anáhuac México Campus Norte, State of Mexico, MEX; 2 Internal Medicine, Facultad de Medicina, Universidad Nacional Autónoma de México, Mexico City, MEX; 3 Internal Medicine, Escuela de Ciencias de la Salud, Universidad Anáhuac Puebla, Puebla, MEX; 4 Internal Medicine, Área Académica de Medicina del Instituto de Ciencias de la Salud, Universidad Autónoma del Estado de Hidalgo, Pachuca, MEX; 5 Internal Medicine, Regional General Hospital 196, Fidel Velazquez Sanchez, Instituto Mexicano del Seguro Social (IMSS), State of Mexico, MEX

**Keywords:** antiphospholipid syndrome, autoimmune diseases and western blot, human immunodeficiency virus, stroke, β2-glycoprotein i

## Abstract

The importance of stroke in young adults lies in the clinical challenge it represents, because of the frequent association with uncommon causes and non-atherosclerotic causes, such as the antiphospholipid syndrome (APS). This last one plays a very important role in strokes in this population, especially in patients with the absence of conventional vascular risk. We report a 34-year-old male adult with no prior history of thrombotic events or cardiovascular risk factors, who started with focal neurological symptoms. Many studies were performed; a simple coronal skull computed tomography scan revealed ischemic infarction in the right parietal lobe, and there was no evidence of significant atheromatous plaque or heart defects. Due to the suspicion of a prothrombotic state, laboratory studies were performed, revealing positivity for lupus anticoagulant (LA) and immunoglobulin (IgG) anticardiolipin antibodies, supporting the diagnosis of primary APS. Further viral serological testing confirmed previously undiagnosed human immunodeficiency virus infection (HIV).

The patient was managed with antiretroviral therapy, statins, antiplatelet, prophylactic antibiotics, and physical rehabilitation, showing significant improvement. This case highlights the importance of the early and accurate etiology diagnosis on a population that may represent a challenge to diagnose, in this case we could see how the stroke was linked with the APS and the HIV, and how timely and appropriate management can significantly influence prognosis and reduce the risk of recurrent prothrombotic events.

## Introduction

Ischemic cerebrovascular events currently represent the third leading cause of death and combined disability worldwide, with an estimated incidence ranging from 7,804,449 to 11,900,000 new cases annually, disproportionately affecting individuals residing in low-income and lower-middle-income countries. These events occur predominantly in male individuals, with an incidence of approximately 102 cases per 100,000 men aged 40-84 years, compared with 82 cases per 100,000 women, among whom a higher incidence is observed in those aged ≥85 years [1,2]. However, ischemic stroke (IS) in young adults (<50 years of aged) accounts for approximately 10-15% of all IS cases between 1990 and 2021, while this population contributes to [3] nearly 16% of all deaths related to subarachnoid hemorrhage, with estimates indication that up to one third of cerebrovascular events in these patients are related to non-atherosclerotic causes [2]. 

The etiologic evaluation of IS in young adults is particularly comprehensive and focuses on identifying uncommon causes, including autoimmune diseases, prothrombotic states, and systemic infections [3]. Furthermore, approximately 20% of cerebrovascular events in young patients have been associated with APS [4]. APS is an autoimmune disorder characterized by the development of both arterial and venous thrombosis, associated with the presence of antiphospholipid antibodies, including anticardiolipin antibodies, anti-β2-glycoprotein I (β2GPI) antibodies, and LA. In this disease, ischemic stroke represents the most common and severe clinical complication, particularly in young adults [3,4].

On the other hand, HIV infection is associated with a higher risk of IS, up to threefold greater compared with the general population, particularly among children and young adults, with the risk increasing up to ninefold in advanced stages of acquired immunodeficiency syndrome [5]. The estimated incidence of cerebrovascular events in patients with HIV is 17.9 per 10,000 person-years, supporting HIV infection as an independent risk factor mediated by chronic inflammation, endothelial dysfunctions, and activation of the coagulation system [6]. This is a case of a young adult with IS as the first manifestation of primary APS, in the context of a recent HIV diagnosis, underscoring the importance of early comprehensive evaluation in young patients without conventional vascular risk factors to identify autoimmune and prothrombotic etiologies and prevent recurrence of IS.

## Case presentation

A 34-year-old male with no family history of autoimmune diseases or thrombotic events and no chronic degenerative diseases began experiencing his current condition on June 9, 2025, at approximately 8:30 a.m. Upon waking up, presenting with sudden onset paresthesia in the upper and lower extremities of the left side of the body, lasting approximately two hours. The initial symptoms were accompanied by muscle stiffness in the left side of the body, generalized weakness, paresthesia in the left side of the face, and predominantly distal dysesthesia, with preserved consciousness and speech. He therefore went to the emergency department, with initial vital signs of blood pressure 126/76 mmHg, respiratory rate 19 breaths per minute, heart rate 88 beats per minute, saturation 94% in ambient air, and temperature 36.2 degrees.

Neurological evaluation of the left lower extremity revealed hyperreflexia, increased muscle tone, and a positive Babinski sign, consistent with left upper motor neuron (UMN) syndrome. He had a strength of 3/5 on the Daniels scale in his left upper limb and 3/5 in his left lower limb, with decreased fine sensitivity in the ipsilateral upper limb. His initial National Institutes of Health Stroke Scale (NIHSS) score was 12 points, corresponding to a moderate neurological deficit, and his Glasgow Coma Scale score was 15.

**Figure 1:**
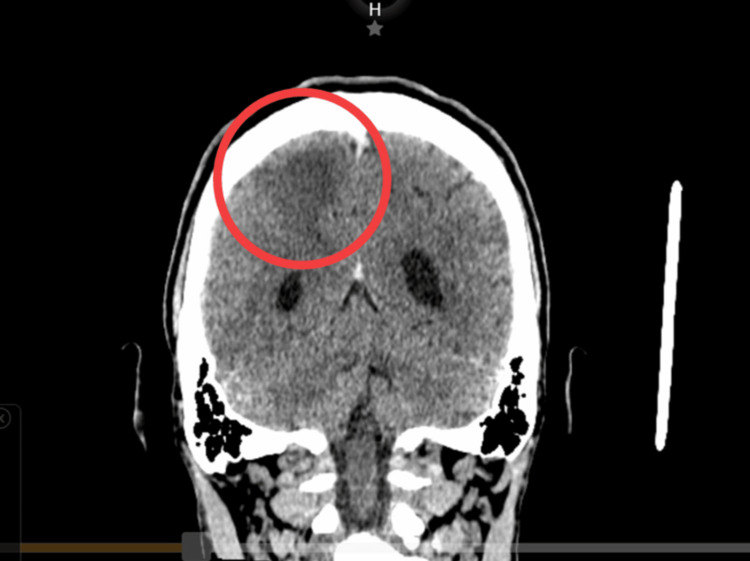
CT scan of the patient's skull (coronal view) Simple coronal skull CT scan with report of ischemic cerebrovascular event in the right parietal lobe, including the semioval center. The red circle indicates the presence of a hypodense lesion consistent with an ischemic cerebrovascular event in the right parietal lobe, including the semioval center.

Due to suspicion of a stroke, a plain computed tomography (CT) scan of the skull was performed, which revealed an ischemic cerebrovascular accident in the right parietal lobe, including the semioval center (Figure 1). Given the time elapsed since the onset of symptoms exceeding 4.5 hours, the patient was not a candidate for intravenous thrombolysis, nor did he meet the imaging or clinical criteria for mechanical thrombectomy. The neurology department expanded its investigations to determine the etiology of the stroke in this young patient. A carotid Doppler ultrasound was requested and showed no significant atheromatous plaques, but did reveal findings consistent with vertebrobasilar insufficiency. The transthoracic echocardiogram showed no septal defects, valvular heart disease, patent foramen ovale, or cardiometabolic sources. Blood tests were uneventful, with a lipid profile showing triglycerides 158 mg/dL, LDL cholesterol 52 mg/dL, and HDL cholesterol 15 mg/dL, and a coagulation profile showing prothrombin time (PT) 12 seconds and partial thromboplastin time (PTT) 28 seconds. 

Given the patient's age, the absence of associated risk factors, and the suspicion of a prothrombotic state, markers for autoimmune diseases were requested, with positive results for lupus anticoagulant and IgG anticardiolipin antibodies (Table 1), which led us to suspect primary antiphospholipid syndrome as the cause of the stroke. Viral serology tests are requested for hepatitis B virus (HBV), hepatitis C virus (HCV), and human immunodeficiency virus (HIV). The HIV test is reactive, so viral load and Western Blot tests are requested for confirmation, confirming the presence of infection with a viral load of 1,117,549 and a CD4 count of 23 cells/µl, which tested positive for human immunodeficiency virus type I (Table 1).

**Table 1:** Laboratory test result June 2025 With positive anticoagulant and IgG anticardiolipin antibody reports suggesting ASP, and the presence of infection with a viral load of 1,117,549 and a CD4 count of 23 cells/µl, the patient tested positive for HIV type I. APS: antiphospholipid syndrome, LA: lupus anticoagulant, HIV: human immunodeficiency virus, DRVVT: Dilute Russell's Viper Venom Time. Ig: Immunoglobulin, CD4: Cluster of Differentiation 4.

Laboratory test	Results	Normal parameters
LA	Positive	Negative
DRVVT Screen	1.43 ratio	0.00 - 1.20 ratio
DRVVT Confirm	1.13 ratio	0.00 - 1.20 ratio
Normalized ratio	1.27 ratio	0.00 - 1.20 ratio
Anti-cardiolipin IgG antibodies	28.5 CU	< 20 CU
Anti-cardiolipin IgM antibodies	5.3 CU	< 20 CU
Anti-Beta 2 Glycoprotein IgG Antibodies	< 6.4 CU	< 20 CU
Anti-Beta 2 Glycoprotein IgM Antibodies	12.3 CU	< 20 CU
Anti-HIV antibodies 1 and 2	77.70 S/U (reactive)	Non-reactive
Viral load for HIV	1117549 copies/m	< 40 copies
CD4 T lymphocytes	23 cells/μL	404-1612 cells/μL

Treatment begins with high-intensity statins, antiplatelet agents (acetylsalicylic acid), anticoagulants based on vitamin K antagonists (VKA), prophylaxis for opportunistic infections, as well as antiretroviral therapy and physical rehabilitation to enable the patient to resume basic and instrumental activities of daily living. Confirmation of antiphospholipid antibody positivity was performed at 12 weeks (Table 2). The INR targets are monitored very closely, as they are currently not on target. Currently progressing well and undergoing multidisciplinary follow-up.

**Table 2:** Laboratory test result October 2025 Confirmation of antiphospholipid antibody positivity was performed at 12 weeks. LA: lupus anticoagulant, DRVVT: Dilute Russell's Viper Venom Time. Ig: Immunoglobulin, INR: International normalized ratio.

Laboratory test	Results	Normal parameters
LA	Positive	Negative
DRVVT Screen	2.62 ratio	0.00 - 1.20 ratio
DRVVT Confirm	1.74 ratio	0.00 - 1.20 ratio
Normalized ratio	1.51 ratio	0.00 - 1.20 ratio
Anti-cardiolipin IgG antibodies	28.1 CU	< 20 CU
Anti-cardiolipin IgM antibodies	5.8 CU	< 20 CU
Anti-Beta 2 Glycoprotein IgG Antibodies	< 6.4 CU	< 20 CU
Anti-Beta 2 Glycoprotein IgM Antibodies	30.5 CU	< 20 CU
INR	1.03	0.8-1.2

## Discussion

The epidemiology of APS in the general population shows an estimated incidence of one to two cases per 100,000 individuals [7], and it is more frequently observed in women. Although the age of diagnosis varies across epidemiological regions, the mean age in the general population ranges from 55 to 59 years in men, while women tend to present a higher incidence at a younger age, around 37 years [8,9]. Notably, patients with APS have a higher mortality rate than the rest of the population, varying across 65% higher than healthy individuals [7]. Additionally, it has been reported that the prevalence of thrombotic events increases with advancing age and is the most common manifestation of APS across all populations, primarily attributed to the presence of antibodies such as antiphospholipids (aPLs) and anticardiolipins (aCLS) [10,11].

In these patients, such antibodies are directed against a neoepitope formed by the binding of the lipid-binding coagulation inhibitor to phospholipids on the cellular membrane named β2-glycoprotein I (β2GPI) [12]. In patients with HIV infection, aPL are frequently detected as a transient phenomenon, usually at low titers and without clinical relevance; However a subset of patients develops persistent antibodies dependent on β2GPI, which are associated with clinically relevant thrombotic manifestations, including arterial events such as ischemic stroke (IS) and transient ischemic attack (TIA) [13]. Moreover, in certain viral acute infections, such as HIV, these antibodies are strongly linked with viral replication [14], also, its presence has been detected likely as a consequence of intense immune system activation. In such contexts, antibodies recognize lipid components of the cell membrane but do not directly participate in the coagulation cascade; rather, their presence appears to reflect heightened antigenic stimulation of the immune response against the present infection [15]. However, in a subset of patients, these antibodies may interfere with anticoagulant pathways by impairing the protein C (PC) and protein S (PS) system, promoting endothelial activation and tipping the hemostatic balance toward a prothrombotic state [16]. Furthermore, as in APS, thrombotic manifestations occur far more frequently in patients with HIV than in those with other viral infections, which supports the hypothesis of a strong immunological dysregulation enhanced between both conditions [13], as in our patient. 

Our case describes a 34-year-old man without vascular risk factors (RF) who presented with a right parietal ischemic stroke (IS) and a moderate focal neurological deficit with a National Institutes of Health Stroke Scale (NIHSS) score of 12, without evidence of significant atherosclerosis, structural heart disease, or a cardioembolic source. This pattern is consistent with that described in other reports of young patients with HIV infection and aPL [17] reported an HIV positive patient with multiple TIAs and a mild IS associated with aCL. Similarly, Gorczyca et al. described recurrent cerebral infarctions as the first clinical manifestation of HIV infection, highlighting that IS may represent an initial presentation in this population [16]. As in our patient, the absence of conventional RF in these cases supported the suspicion of an underlying prothrombotic etiology [16,17].

As mentioned before, patients with HIV infection tend to present a transitory elevation of aPL and aCL, and it has been documented in several case reports in patients with HIV infection presenting with cerebral ischemic events [13,16,17]. In our patient, persistent positivity for lupus anticoagulant (LA) and IgG aCL, confirmed at 12 weeks and associated with IS, allowed the diagnosis of APS to be established. A distinctive aspect of our case is the coexistence of a high viral load (VL) and profound immunosuppression. To date, no studies or case reports of IS associated with HIV infection and APS have systematically documented VL levels and CD4 counts at the time of stroke onset. Nevertheless, high VL has been associated with an increased frequency of clinical complications, including cardiovascular (CV) and thrombotic events, as well as increased production of proinflammatory cytokines and activation of the coagulation system [13,18]. 

Despite this prothrombotic state, conventional coagulation tests, including prothrombin time (PT) and activated partial thromboplastin time (aPTT), remained within normal ranges. This can be explained by the fact that, in both HIV infection and APS, arterial thrombosis is primarily driven by endothelial and platelet activation, increased tissue factor (TF) expression, release of procoagulant microparticles (MPs), and reduction of natural anticoagulants such as PC and PS. These mechanisms are not evaluated by standard coagulation assays, which measure only plasma coagulation factor activity [19]. Therefore, HIV induced endothelial dysfunction and the proinflammatory state associated with high VL may promote arterial thrombosis even in the absence of PT or aPTT abnormalities. This explains why IS may occur acutely in young patients without apparent comorbidities, as observed in our patient [5]. Similar to other reported cases, the management instituted in our patient, including anticoagulation with VKA, standard IS treatment, initiation of antiretroviral therapy (ART), and opportunistic infections (OI) prophylaxis, represents the recommended therapeutic approach to reduce the risk of thrombotic recurrence in patients with APS associated with HIV infection.

## Conclusions

Stroke in young adults should prompt a comprehensive approach to rule out secondary causes. The coexistence of primary antiphospholipid syndrome and HIV, as observed in this patient, constitutes a diagnostic and therapeutic challenge that directly impacts prognosis and the prevention of thrombotic recurrences.
